# Nutraceutical Potential and Food Safety of Fructose in Soda and Diet Beverages

**DOI:** 10.3390/foods14040648

**Published:** 2025-02-14

**Authors:** Marcos Mateo-Fernández, Pilar Alves-Martínez, Mercedes Del Río-Celestino, Rafael Font, Tania Merinas-Amo, Ángeles Alonso-Moraga

**Affiliations:** 1 Department of Genetics, University of Córdoba, 14071 Córdoba, Spain; marcosmatfer@gmail.com (M.M.-F.); piluca.alves@gmail.com (P.A.-M.); tania.meram@gmail.com (T.M.-A.); ge1almoa@uco.es (Á.A.-M.); 2 Agri-Food Laboratory, CAGPDS, Avd. Menéndez Pidal, s/n, 14080 Córdoba, Spain; rafaelm.font@juntadeandalucia.es

**Keywords:** fructose, Diet Coke, Pepsi, food safety, nutraceutical potential

## Abstract

Fructose has been considered as an additive from soda beverages. For the approval of new additives or to extend the usage of an approved one, it is necessary to conduct toxicological studies in order to evaluate the DNA damage induced by these compounds. Our study is based on evaluating the safety and the nutraceutical potential of Fructose (FRU), a soda cola beverage (Pepsi-cola, PEP), and a diet soda cola (Diet Coke, DCC), characterizing the DNA changes induced in the *Drosophila melanogaster* organism model and in the human leukemia HL-60 cells performing different assays. Our results showed neither the toxicity nor mutagenic activity of FRU, PEP, and DCC in *Drosophila melanogaster*, while only PEP exhibited protective effects in the antitoxity assay, showing an 80% survival rate in combined treatments. FRU, but not PEP, enhanced lifespan parameters by up to 23 more days at the 5 mg/mL concentration. All three substances exhibited chemopreventive properties in some of the checkpoints carried out related to clastogenicity and methylation patterns in HL-60 cells. In conclusion, the tested compounds were safe at tested concentrations in *Drosophila* and showed moderate chemopreventive activity.

## 1. Introduction

It is known that all somatic cells from an individual organism contain the same genome, but genes are not all expressed in a cell due to the well-known genetic process called selective expression. Epigenetics are one of the main mechanisms of regulation which consist of inherited changes in gene expression produced by environmental factors and not directly related to the alteration of DNA coding sequence. Chromatin is modulated by these epigenetic marks [1].

Nutrition is associated with epigenetic modifications and closely related to an increase in cancer incidence since the 1960s. Regarding cancer, it is also an epigenetic disease in which apoptosis is controlled by cancer mechanisms [2]. Conversely, diet restriction could be involved in anti-inflammatory processes and thus, could extend lifespan [3]. Dietary patterns, not only individual compounds, also influence the epigenome throughout life. Therefore, it is important to holistically study these substances in foods and beverages [4]. In addition, reactive oxygen species (ROS) are produced by all aerobic cells and play a vital role in aging and degenerative disease. The influence of ROS and antioxidants on gene expression and H_2_O_2_ has been used as a concurrent oxidative stimulus [5].

The classification of FRU as a nutraceutical is complex. While FRU is a natural sugar found in fruits and honey, its potential health benefits depend on the context and amount consumed. FRU alone is not typically regarded as a nutraceutical because excessive intake, particularly from processed foods and sweeteners, is associated with adverse health outcomes like obesity and metabolic syndrome. However, in controlled amounts and as part of whole foods like fruits or beverages, FRU might contribute to health benefits due to its integration with other bioactive compounds. Nutraceutical effects often arise from such compounds, including vitamins, polyphenols, and fiber, rather than fructose itself. Research highlights the potential of specific diets incorporating balanced fructose sources to modulate gene expression favorably and improve metabolic health [6].

FRU has been linked to epigenetic changes and the modulation of gene expression. Studies have shown that high fructose intake can influence DNA methylation patterns, a critical epigenetic mechanism regulating gene activity. For instance, fructose consumption has been associated with the altered expression of genes related to inflammation and metabolism through changes in methylation marks and histone acetylation. This suggests that fructose can impact cellular function at the genomic level, potentially contributing to metabolic disorders when consumed in excessive amounts. In experimental models, such as mice fed a high-fructose diet, epigenetic effects were observed in liver cells, where DNA methylation changes were linked to the activation of genes promoting fat accumulation and inflammation. These findings provide insights into how fructose can influence disease pathways through epigenetic regulation [7,8].

*Drosophila melanogaster* has been widely recognized as a reliable model for translational biomedicine purposes as it has shown its high specificity, with more than 80% of genes linked to human genetic disease homologs [9,10]. A commonly employed in vivo system of *Drosophila* for genetic studies is the Somatic Mutation and Recombination Test (SMART). This test involves genetic alterations that are induced in the imaginal disc cells of larvae, which, following clonal expansion and metamorphosis, are phenotypically expressed in the adult tissues. It has been demonstrated that the SMART is effective in detecting the crucial genotoxic/antigenotoxic activity of single molecules and complex mixtures with most structural types and mutagenic activity models as it allows to classify them as possible carcinogens or anticarcinogens [11,12,13].

In addition, *Drosophila* serves as a reliable biological system for aging studies, as adult flies exhibit numerous similarities to cellular senescence observed in mammals [14]. This characteristic has led to the frequent use of this model to investigate the relationship between nutrient metabolism and aging mechanisms [15]. Significant contributions to this area are expected in the future [16].

In parallel, cytotoxicity assays conducted in vitro are commonly employed to evaluate the chemopreventive potential of various compounds [17]. Given the high toxicity and lack of specificity in cancer therapies, an alternative strategy might involve using agents capable of inducing differentiation in cancerous cells [18]. The HL-60 human leukemia cell line is frequently utilized to assess the ability of compounds to induce cell differentiation and proapoptotic mechanisms. Compounds that demonstrate these properties could be regarded as potential chemopreventive agents [19,20].

DNA methylation is a crucial epigenetic mark that is involved in the transcriptional silencing of genes and plays an essential role in development and in defending the genome against transposable elements [21]. The methylation status of transposable sequences is particularly important for understanding the overall DNA methylation landscape. An effective strategy for cancer prevention might involve the prevention or reversal of the hypermethylation-induced inactivation of tumor suppressor genes or gene receptors through the use of DNA methyltransferase (DNMT) inhibitors [22].

Taking all of the above into account, our study is based on evaluating safety first, and the nutraceutical potential of Fructose (FRU), Pepsi-cola (PEP), and Diet Coke (DCC), characterizing the DNA changes induced in the *D. melanogaster* organism model and human leukaemia HL-60 cells performing different assays. Toxicity, genotoxicity, and comet assays were carried out in order to evaluate the food safety of the tested compounds. Antitoxicity, antigenotoxicity, cytotoxicity, and methylation status were used to determine the antioxidants, chemoprevention, and DNA modulation potential related to nutraceutical characterization, as well as the life extension assay. The nutraceutical value of any substance can be determined by performing genotoxicologic bioassays [23,24].

## 2. Materials and Methods

### 2.1. Samples

Fructose (FRU), which was selected as a primary component of sugar-containing beverages, such as Pepsi-Cola (PEP), was obtained from ApplichemPanreac (Cat. No. 57-48-7). FRU (180.16 g/mol) is a well-known reducing ketohexose used as a food additive and considered as a nutritional sweetener in cola beverages. The PEP and Diet Coke (DCC) samples used in this study were purchased from a local market in Córdoba, Spain. After purchasing, they were lyophilized. The concentration range of FRU tested had been previously described by Ventura, et al. [25]. To ensure comparability with a human’s daily consumption of PEP (2 L of cola per day for a 70 kg individual), the concentrations of the beverage used in the various assays were calculated based on the average daily food intake of *D. melanogaster* (1 mg/day) and the average body weight of *D. melanogaster* individuals (1 mg) [26].

### 2.2. In Vivo Fly Stocks

Two strains of *D. melanogaster*, each with distinct genetic markers affecting the wing-hair phenotype, were utilized in the study: (i) *mwh*/*mwh*, which carries the recessive mutation *mwh* (multiple wing hairs) [27], and (ii) *flr^3^*/*In (3LR) TM3*, *rip^p^se^p^ bx^34e^e^s^Bd^S^*, where the *flr^3^* (*flare*) [28] marker represents a homozygous recessive lethal mutation. This mutation is viable in homozygous somatic cells once the larvae begin developing, leading to the production of deformed trichomonas. Additionally, the flare marker (*flr^3^*) is maintained through the chromosomal balancer *TM3*, *Bd^s^* (*flr^3^*/*TM3*, *Bd^s^*). Detailed information regarding these genetic markers can be found in Lindsley and Zimm [29].

### 2.3. In Vitro Cell Culture Conditions

Human promyelocytic leukemia (HL-60) cells were used and maintained in the same conditions as previously published by the authors [30].

### 2.4. In Vivo Safety Studies

#### 2.4.1. Toxicity Assays

Toxicity was assessed following our standard protocols as described by Mateo-Fernández, et al. [13]. Additionally, a lethal dose 50 (LD_50_) was determined to evaluate the toxicity of the compounds.

#### 2.4.2. Genotoxicity Assay

Genotoxicity assays were conducted following the standard wing spot test procedure [9]. Briefly, transheterozygous larvae for the *mwh* and *flr^3^* genes were obtained by crossing four-day-old virgin *flr^3^* females with *mwh* males at a 2:1 ratio. Four days after fertilization, the females were allowed to lay eggs in fresh yeast medium (25 g of yeast and 4 mL of sterile distilled water) for 8 h to obtain synchronized larvae. After 72 h, larvae were collected, washed with distilled water, and grouped into clusters of 100 individuals. Each group was fed a mixture of 0.85 g *Drosophila* Instant Medium (Formula 4–24, Carolina Biological Supply, Burlington, NC, USA) and 4 mL of water, supplemented with the tested compounds at the highest and second lowest concentrations from the toxicity assays, as well as negative (H_2_O) and positive (0.15 M H_2_O_2_) controls, until pupae hatched (10–12 days). Adult flies were collected and wings were mounted on slides using Faure’s solution. Mutant spots were scored on both the dorsal and ventral surfaces of the wings using a bright light microscope at 400× magnification.

The frequencies of each type of mutant clone (single, large, or twin spots) per wing were compared to the negative control, and the results were analyzed using the Kastenbaum and Bowman binomial test [31]. Inconclusive results from the binomial test were further analyzed using the Mann–Whitney and Wilcoxon U-test [12,32].

### 2.5. In Vivo Evaluation of Nutraceutical Potential

#### 2.5.1. Antitoxicity Assay

Experiments were performed following the methodology of Tasset, et al. [33], including a positive control consisting of 0.15 M H_2_O_2_. Significances were assessed using the non-parametric Chi-square test [13].

#### 2.5.2. Antigenotoxicity Assay

Antigenotoxicity tests were performed, following the method described by Anter, et al. [11] and the antigenotoxic capacity was measured by the Inhibition Percentage (IP) formula. The IP for the combined treatments were calculated when appropriate according to Abraham and Singh [34]:

IP = [(genotoxin alone − combined treatment)/genotoxin alone] × 100

Inconclusive results from the Kastembaum–Bowman binomial test were also resolved using the Mann–Whitney U-test [12,32].

#### 2.5.3. Chronic Treatments: Lifespan and Healthspan Assays

To ensure consistency across all in vivo assays, an F_1_ progeny from the *mwh* and *flr^3^* parental strains was used, produced by a 24-h egg-laying period in yeast for all longevity trials. The same concentrations used in the toxicity/antitoxicity experiments were also tested in the lifespan assays. These assays were conducted at 25 °C, following the procedure outlined by Fernandez-Bedmar, et al. [35]. Briefly, transheterozygous larvae, aged 72 ± 12 h, were synchronized, washed with distilled water, collected, and transferred in groups of 100 into test vials. These vials contained 0.85 g of *Drosophila* Instant Medium and 4 mL of the different concentrations of the compound being tested. Emerged adults from the pupae were collected under CO_2_ anesthesia and placed in groups of 25 individuals of the same sex into sterile vials containing 0.21 g *Drosophila* Instant Medium and 1 mL of different concentrations of FRU, PEP, and DCC when appropriate.

The flies were subjected to chronic treatment throughout their lifespan. The number of surviving individuals was determined twice a week. The survival data were statistically analyzed using SPSS 19.0 (SPSS, Inc., Chicago, IL, USA), with the Kaplan–Meier method applied to calculate survival curves. The significance of these curves was assessed using the Log-Rank test (Mantel–Cox).

### 2.6. In Vitro Evaluation of Nutraceutical Potential

#### 2.6.1. Cytotoxicity Assay

The impact of the tested compounds on cell viability was assessed using the trypan blue exclusion assay, following standard procedures [11]. The curves were generated using Microsoft Excel. Finally, the IC_50_ value was estimated.

#### 2.6.2. DNA Fragmentation Status

The ability of our compound to induce DNA fragmentation was determined as described by Anter, et al. [36]. Briefly, 10^6^ HL-60 cells were co-cultured with five different concentrations of FRU, PEP, and DCC (as selected in the toxicity assays) for 5 h. The apoptosis process can be determined by the appearance of internucleosomal DNA fragments that are multiples of 200 base pairs.

#### 2.6.3. Clastogenicity: Single-Cell Gel Electrophoresis (SCGE, Comet Assay)

DNA integrity was evaluated by SCGE following the protocol outlined by Mateo-Fernández, et al. [13], undergoing treatment, washes, lysis, electrophoresis, neutralization, and propidium iodide staining. OpenComet plugging in ImageJ (NIH) software was used to analyze the Tail Moment (TM) [37].

#### 2.6.4. Methylation Status of HL-60 Cells

The assay to assess DNA methylation status was performed following the protocol outlined by Merinas-Amo, et al. [12]. HL-60 cells were exposed to several concentrations of FRU (2.25 mg/mL and 72 mg/mL), PEP (3.43 mg/mL and 110 mg/mL), or DCC (0.09 and 3 mg/mL) for a period of 5 h. DNA was subsequently extracted using the same method as in the DNA fragmentation assay. The extracted DNA was treated with bisulphite, utilizing the EZ DNA Methylation-Gold Kit (Zymo Research, Irvine, CA, USA). Fluorescence-based real-time quantitative Methylation-Specific PCR (qMSP) was conducted using the primer indicated in Table 1.

The CT (cycle threshold) values were obtained for each qMSP, and data normalization was carried out using the ΔΔCT method, as previously described by Nikolaidis, et al. [38] and Liloglou, et al. [39]. One-way ANOVA and post hoc Tukey’s test were applied to analyze the differences between the tested compounds, repetitive elements, and concentrations.

**Table 1:** Primer information [40].

Primer	Forward Primer Sequence 5′ to 3′ (N)	Reverse Primer Sequence 5′ to 3′ (N)
ALU-C4	GGTTAGGTATAGTGGTTTATATTTGTAATTTTAGTA (−36)	ATTAACTAAACTAATCTTAAACTCCTAACCTCA (−33)
ALUM1	ATTATGTTAGTTAGGATGGTTTCGATTTT (−29)	CAATCGACCGAACGCGA (−17)
LINE-1-M1	GGACGTATTTGGAAAATCGGG (−21)	AATCTCGCGATACGCCGTT (−19)
SAT-α-M1	TGATGGAGTATTTTTAAAATATACGTTTTGTAGT (−34)	AATTCTAAAAATATTCCTCTTCAATTACGTAAA (−33)

ALU (short interspersed nuclear element, as SINE and Alu-C4 housekeeping sequence). LINE (Long Interspersed Nuclear Element M1). Sat-α (Satellite alpha DNA).

## 3. Results

### 3.1. In Vivo Assays

Table 2 shows the toxicity and antitoxicity results obtained in this study. These results revealed that although PEP was significantly toxic at most tested concentrations, the LD_50_ was not reached in any concentrations. Regarding FRU and DCC, they were not toxic at any tested concentrations. According to the antitoxicity assay, antioxidant properties were not found in any compounds and tested concentrations, except for the second-highest concentration of FRU which significantly showed antitoxic effects in *D. melanogaster*.

SMART results are depicted in Table 3. The concurrent positive control showed significant differences with respect to the negative control using the Kastenbaum–Bowman statistical test providing a mutation rate per wing of 0.425 against 0.195, respectively. This result validates the accuracy of the assay. In regard to genotoxicity, both FRU and DCC showed inconclusive results, and they were solved by applying the Mann–Whitney test; they were not genotoxic compounds. PEP resulted in non-genotoxic compounds since negative results were obtained in the Kastenbaum–Bowman test. According to the antigenotoxicity assay, combined treatments of PEP and hydrogen peroxide showed positive (*) results, which means that there are significant differences between PEP and the positive control. Similarly, the lowest concentration of DCC also provided positive results, like PEP. The IP was calculated since positive results were found. The lowest and the highest concentration assayed of PEP have protected DNA by 68% and 76.5%, respectively. The IP obtained in the lowest concentration of DCC was 76.4%. Nevertheless, the IP from FRU was not needed to ascertain as the Kastenbaum–Bowman test provided inconclusive results and they were solved using U Mann–Whitney test, resulting in negative results. Therefore, FRU did not exert any DNA protection against hydrogen peroxide.

**Figure 1:**
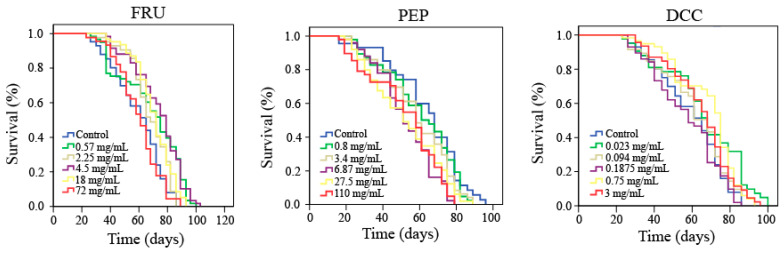
Survival curves obtained from the Log-Rank test evaluating the effect of FRU, PEP, and DCC supplementation on the lifespan of *D. melanogaster*.

Lifespan and healthspan results are plotted in Figure 1 and Table 4, which reported that FRU significantly increased both the lifespan (more than 14%) and healthspan (35% on average) of *Drosophila* when flies are treated with 0.57, 2.25, and 18 mg/mL. DCC also significantly increased lifespan of *Drosophila* when flies are treated with 0.75 mg/mL. On the contrary, when *Drosophila* is treated with the three-highest assayed concentrations of PEP, the life expectancy of this model organism decreased by roughly 18%.

### 3.2. In Vitro Assays

**Figure 2:**
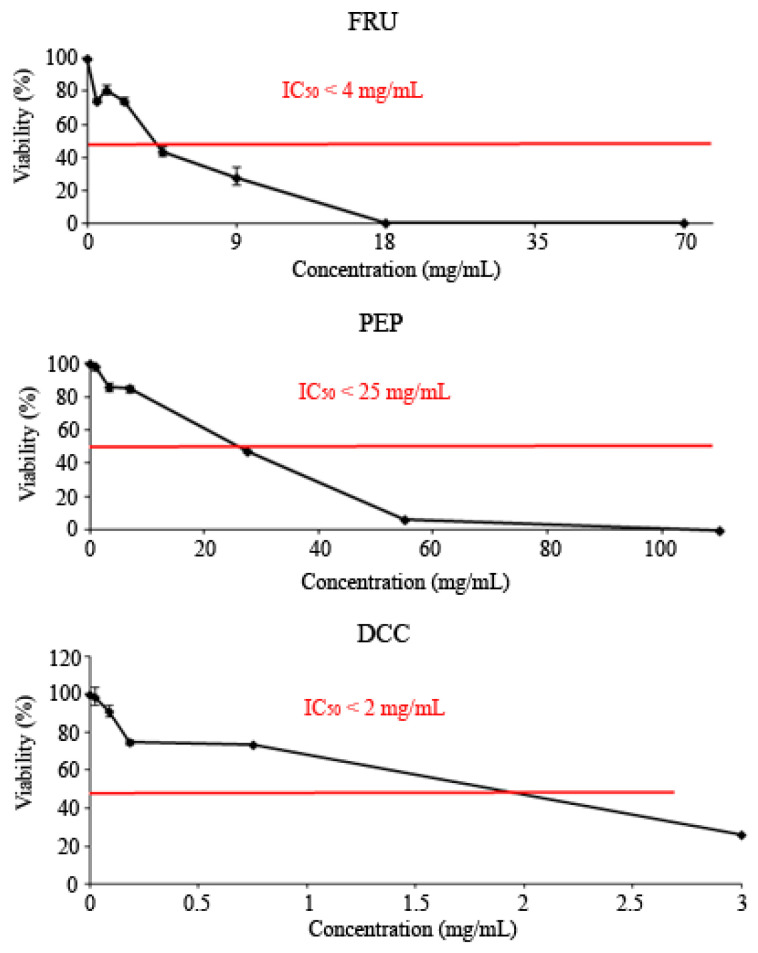
Viability of HL-60 cells treated with FRU, PEP, and DCC for 72 h. Each point represents the percentage of viability with respect to the mean control ± SD of three independent experiments.

All tested compounds showed cytotoxic effects against the HL-60 leukemia cell line, as shown in Figure 2. The IC_50_ was reached at roughly 3 mg/mL, 25 mg/mL, and 2 mg/mL for the FRU, PEP, and DCC treatments, respectively.

**Figure 3:**
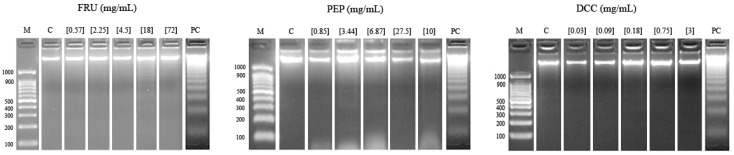
Internucleosomal DNA fragmentation after 5 h of HL-60 cells treated with FRU, PEP, and DCC. Letters M and C are the weight size marker and negative control (RPMI), respectively, and lyophilized blond beer (62.5 mg/mL) was used as a routine positive control (PC).

Figure 3 shows the results of the internucleosomal DNA fragmentation test. The result obtained in this assay reported that none of the tested compounds were able to modify the DNA integrity of the HL-60 cell line since a ladder pattern is not induced, except for 18 mg/mL FRU and 3.44 mg/mL PEP, to some extent.

**Figure 4:**
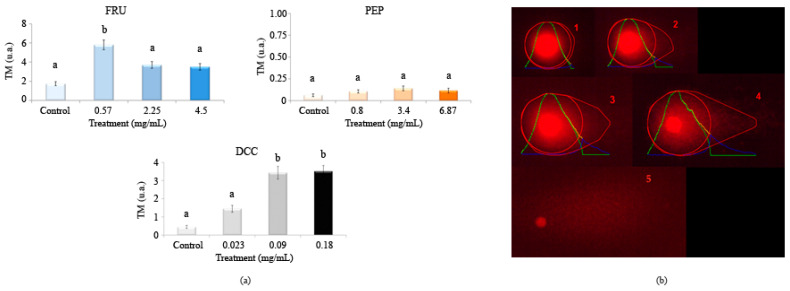
Alkaline comet assay (pH > 13) of HL-60 cells after 5 h of treatment with different concentrations of FRU, PEP, and DCC. (**a**) DNA migration is reported as mean TM. The graph shows mean TM values and standard errors. Statistical differences were analyzed, applying one-way ANOVA and post hoc Tukey’s test. TM: Tail Moment. (**b**) Representative results after applying comet assay software are depicted: (1) TM = 0.17 (typical control result); (2) TM = 3.45; (3) TM = 6.12; (4) TM = 16.4; (5) hedgehog pattern of apoptosis, which was not found in our results. a,b: statistical differences between treatments.

Figure 4 shows the results in the single cell gel electrophoresis test or comet assay evaluating the genomic integrity of HL-60 cells treated with different concentrations of FRU, PEP, and DCC. According to this assay, DCC significantly increased the TM value with respect to the concurrent control, inducing DNA damage in the HL-60 cell line at most tested concentrations. No DNA damage was observed at any concentrations of PEP. However, 18 mg/mL FRU induced internucleosomal DNA fragmentation in the HL-60 cell line. The concentrations used in this SCGE assay were determined according to the results obtained in the previous cytotoxicity assay.

**Figure 5:**
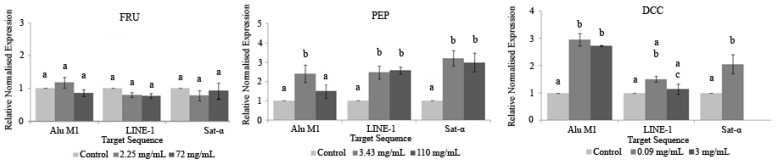
Relative normalized expression data of each repetitive element. Different letters are associated with different means applying the one-way ANOVA test and post hoc Tuckey’s test.

Figure 5 shows the relative normalized methylation status (RMS) of the three repetitive sequences (LINE-1, Alu M1, and Sat-α) in the HL-60 cell line treated with our compounds tested in this study. PEP and DCC generally hypermethylated AluM1, LINE-1, and Satellite-α repetitive elements, except for the LINE-1 when cells are treated with DCC, for which the values obtained were similar to the concurrent control. The cytotoxic effect of the beverages is the reason why 3 mg/mL DCC is not represented in Figure 5 due to the lack of cells to conduct the assay.

## 4. Discussion

The definition of a food additive has changed across time. According to the Food Protection Committee of the Food and Nutrition Board, food additives may be defined as follows: “a substance or mixture of substances, other than a basic foodstuff, which is present in a food as a result of any aspect of production, processing, storage, or packaging. The term does not include chance contaminants”. Today, it is defined by Codex Alimentarius as “any substance not normally consumed as a food by itself and not normally used as a typical ingredient of the food, whether or not it has nutritive value”. This definition was proposed in 1995 by the joint panel, which comprised the Food and Agriculture Organization (FAO) of the United Nations and the World Health Organization (WHO), and was revised over the subsequent years.

However, this classification is not clear. Scientists report that nutritional compounds should not be considered as additives due to the fact that they yield nutritional value to the food where they are added so they should be named as enrichments to foodstuffs. In addition, their consumption has increased due to functional and nutraceutical activities; therefore, the scientific interest from these enrichments to food has increased. Nutritional enrichments are natural, for instance, vitamins, amino acids, fibers, fatty acids and polyphenols, among others [42].

In our study, FRU has been considered as an additive from soda beverages, and it could have nutraceutical properties in the appropriate amounts and not only as flavor enhancers. Some procedures have to be performed in order to approve new additives or extend the usage of an approved one within the EU. One of these procedures consists of toxicological studies, which encompass in vitro and in vivo assays. The latter includes metabolism/toxicokinetics, acute subchronic and chronic toxicity, as well as genotoxicity, carcinogenicity, reproduction, absorption, developmental toxicity, immunotoxicity, and hypersensitivity/allergy in various model organisms [43]. Updated assays related to genotoxicology, toxicology, and nutraceutical potential in a broad sense have been demonstrated to be necessary in order to report new corpus data to elucidate the additives’ safety. The results obtained all over the world will allow the major regulators of food additives to establish new rules associated with them. PepsiCo has been promoting diet beverages due to the high content of sugar in the soda beverages and the health risk they could cause; however, there is no scientific evidence about this risk. Once more, the atmosphere about soda and additives security is not clear [44].

### 4.1. In Vivo Safety Studies

The present work evaluates the safety of an additive, FRU, and two soft drinks, PEP and DCC, in in vivo toxicity and genotoxicity assays using the eukaryotic *Drosophila* model.

Despite PEP and DCC being highly consumed beverages over the world, not much information about their toxicity and genotoxicity has been reported. FRU is reported to promote lipogenesis and cause insulin resistance, a prelude to diabetes, and obesity. However, FRU could be considered as a nutraceutical detoxicating drug-induced liver damage [45].

FRU is one of the main components used in soft drinks. It has been shown that FRU is less satiating and more lipogenic than glucose [46]. In addition, a decreasing level of insulin and leptine and an increasing level of ghreline have been related to a high consumption of FRU [47].

In this study, PEP was toxic at the three highest concentrations in *D. melanogaster*. Nevertheless, the LD_50_ of PEP was not found at any concentration; therefore, the beverage could be considered as non-toxic, as related in the report of MacKenzie, et al. [48] where they used color caramel from soda beverages

FRU and DCC did not exert any effect on toxicity in *D. melanogaster* and this result is not in agreement with that obtained by Hwang, et al. [49] and Nyby, et al. [50], who reported that a high consumption of FRU triggered an insulin-resistance disorder and other abnormalities in rats, and even provoked cirrhosis [51]. FRU has been considered as a substance that increases the oxidative stress level, causing toxicity in rats [52,53]. Globally, FRU is considered as a toxin that induces liver toxicity, metabolic disorders, such as diabetes or obesity, and a cancerous molecule [54]. FRU also induced a metabolic syndrome in male Wisar rats, provoking a toxic effect on the renal tubule function [55]. In addition, cardiovascular toxicity was also found in male Wistar albino rats fed with FRU, and supplementation with selenium is needed since it possesses anti-inflammatory and antioxidant effects attenuating the FRU-induced toxicity [56]. FRU was also reported to be toxic in male mice with disorders in the brain and muscles [57]. More toxic results from FRU were found in female Wisar rats fed with FRU, provoking deteriorated ovarian, showing reproductive toxicity [58]. DCC was demonstrated to be toxic in the rat cerebellum [59,60], and DCC (2 mL/day) also induced degenerative changes in the cerebellar cortex in albino rats [61]. In addition, DCC has been used to dissolve the distal intestinal obstruction in cystic fibrosis patients when applied via colonoscopy [62]. More recently, studies reported that DCC induced histopathological changes in the hippocampus of male albino rats [63]. PEP could negatively affect reproductive success in mice and rabbits, causing toxic damage in tissues [64,65].

Food genotoxicologic assays have been widely used over time in order to evaluate the healthy properties of diet before being considered as nutraceutical substances [23,24]. Nowadays, there is a great deal of scientific evidence based on nutraceutics, supporting the fact that consuming food promotes health. Our genotoxicity assays showed that FRU, PEP, and DCC were non-genotoxic at any tested concentrations. However, our FRU results are not in agreement with those obtained by Hansen, et al. [66], who found it genotoxic in rats, increasing the mutation rate. In addition, 1.6 and 25 mM FRU resulted in being genotoxic in the Chinese hamster in treating ovarian cells. Furthermore, FRU was mutagenic in *S. typhimurium* test and human lymphocytes cells [67,68]. In addition, FRU resulted in genotoxicity in pregnant Swiss female mice, affecting the peripheral tissues [69]. Contrarily, FRU was not found to be genotoxic in rats [70]. The highest concentration of sucrose (50 mM) tested in the *Drosophila* SMART assay induced significant increases in spot frequency and the same concentration of sucrose was also genotoxic in the comet assay using DNA tail% in hemocytes of *D. melanogaster* [71].

The lack of genotoxicity observed in *Drosophila* for all the tested compounds confirmed their safety. We hypothesized that the toxicity observed in our compounds may be induced either by a different pathway other than the genotoxic one, or it may be affecting the different genes to be used in this assay.

### 4.2. Evaluation of Nutraceutical Potential

The nutraceuticals area is a growing sector in nutrition to prevent diseases or treat various types of cancer, among other healthcare benefits. The scientific research guesses that the improved safety and potential effects of newly developed nutraceutical products will further stimulate the investments in the future [72]. The present study performs an evaluation of the nutraceutical potential of FRU, PEP, and DCC by carrying out in vivo antitoxicity and lifespan assays as well as in vitro cytotoxicity, internucleosomal fragmentation, single and double DNA strands breaks, and the modulation of methylation patterns in the HL-60 leukaemia cell model.

The antitoxicity results showed that globally, FRU, PEP, and DCC were not antioxidant compounds since they were not able to revert the damage caused by hydrogen peroxide in *D. melanogaster*, except for 100 mM FRU, which did exert a protective effect. CelecandBehuliak [73] supported the present study since they did not find any antioxidant activity of PEP in rats. In addition, PEP consumption in rats induced vitamin D deficiency, which is associated with an imbalance in antioxidant mechanisms against oxidative stress, which could explain the malfunction of the liver and kidney [74]. This lack of antioxidant effects could be due to the fact that soda drinks down regulate the expression of antioxidants glutathione reductase [75]. However, our results do not fit those of Hong, et al. [76], who regarded soda drinks as antioxidant beverages in a chemical assay, neither those of Mateo-Fernández, et al. [13], who finds antioxidant activity in other cola beverages in *D. melanogaster*.

Regarding FRU, the lack of antioxidant properties generally demonstrated by FRU could be in agreement with the reports that this compound increases the oxidative stress. Feng, et al. [77] reported that 15 mM FRU was not able to protect hepatocytes against hydrogen peroxide. However, Nieminen, et al. [78] demonstrated 20 mM FRU to be an antioxidant in hepatocytes. FRU (4%) seemed to decrease ATP levels and increase AMP in males of *D. melanogaster*, causing an increase in ROS, which triggers defense mechanisms synthesizing superoxide dismutase enzymes. When these enzymes are synthesized, an increase in hydrogen peroxide levels is triggered [79]. However, uric acid is synthesized when the levels of FRU are low, which results in antioxidant activity. Nevertheless, 10% FRU inhibits the effect of superoxide dismutase, and the protective effect is not possible [80]. FRU triggers the browning reaction, and it is demonstrated to be an antimutagenic *S. typhimurium* test [81], which is not in agreement with our results since FRU did not exert DNA protection against hydrogen peroxide; thus, the antigenotoxic effect of PEP and DCC could be due to the color caramel that shows similar IP [30]. Therefore, the antioxidant and antimutagenic activity of FRU in *D. melanogaster* is highly related to the concentration used and the metabolic pathway triggered.

As far as is known, there is not any scientific information about PEP and DCC evaluating the aging and lifespan modulation by these substances. Lifespan assays showed that some concentrations of PEP and DCC (0.75 mg/mL) significantly decreased the life expectancy of *D. melanogaster*, whereas FRU significantly increased the longevity of this fly to some extent. Furthermore, neither PEP nor DCC significantly differed from their concurrent control when the parameter to be evaluated is the quality of life. Conversely, FRU significantly increased the healthspan of *Drosophila*.

Soft drinks can trigger diabetes and obesity in humans; Shah, et al. [82]. Thus, it would be easy to think that these types of beverages could decrease the quality of life, but there is no scientific evidence of this supposition in humans [45]. On the other hand, cola beverages have demonstrated to be anti-ageing agents in *D. melanogaster* [13]. Caffeine content could be responsible for the reduction in lifespan [83] since fructose and glucose did not shorten the lifespan of *D. melanogaster* [80]. Our results showed that FRU significantly increased the life expectancy and the quality of life of *D. melanogaster* at 2.25, 4.5, and 18 mg/mL FRU), which could be related to those obtained by Driver and Cosopodiotis [84], who reported that the mean longevity exerted by 514 mM FRU was 44 days in *Drosophila*. These results suggest a negative-dose dependent tendency in relation to the lifespan. This finding may be supported by the fact that caloric restriction is related to an increase in life expectancy [85].

Regarding in vitro assays, the aim of the present trial was to determine the potential chemopreventive and genotoxic effect of FR, PEP, and DCC on a human cancer cell model (HL-60 cell line) by performing cytotoxicity, DNA fragmentation, SCGE, and epigenetic modulation assays.

All tested compounds were cytotoxic, reaching the IC_50_. These results are congruent with those obtained by other researchers, since cola beverages have been demonstrated to be cytotoxic [86,87], who reported that PEP showed cytotoxic activity on root meristems of the experimental model *Allium cepa*. Other cola beverages, like Coca-Cola, have been reported as cytotoxic using the HL-60 cell line [13]. Kapicioglu, et al. [88] determined the ability of cola drinks to inhibit the proliferation of gastric mucosal cells, although they were not cancerous. Our results are also in agreement with those obtained by Düsman, et al. [89] who demonstrated that PEP was not cytotoxic in the bone marrow cells of Wistar rats. In addition, cola drinks did not differ from their concurrent control using CACO-2 cells [90]. Conversely, the Coca-Cola beverage induced an increase in fibroblast proliferation, probably due to the sugar content, which could trigger a carcinogenic process; however, the proliferation rate increase depends on the place where the beverage was bought [91]. DCC consumption has been related to a decrease insuffering from pancreatic cancer [92]. Besides, 1 mM FRU inhibits the cell growth of hepatocytes although 10–15 mM FRU protects them [77,78]. In cancer cells, 0.8 M FRU was demonstrated to be chemopreventive [93]. An excess of FRU enhanced cytotoxicity in mice by activating ROS production and causing necrosis in hepatocytes [94]. Besides, FRU intake was associated with some kind of cancer due to the deregulation of GLUT5 expression in a cancerous cell line [95,96]. Our findings suggest that FRU could be responsible for the chemoprevention observed in PEP among other molecules.

A DNA fragmentation test and comet assay were performed in order to evaluate the clastogenic potential of FRU, PEP, and DCC on the HL-60 cell line. The degradation of genomic DNA into internucleosomal fragments was proposed as a major mechanism affecting cancer cell apoptosis. Figure 3 shows that the typical ladder pattern is shown when cells are treated with 18 mg/mL FRU. However, the pattern mentioned has not generally appreciated by any of the tested compounds; thus, apoptosis is not induced. It is known that substances, such as cola beverages, induced apoptosis in different research [12,13]. FRU (20 mM) has been shown to inhibit apoptosis in hepatocytes after oxidative damage due to a decrease in ROS [97], which is congruent with our FRU results. Paradoxically, high-grade cancers are generally associated with high constitutive levels of apoptosis. In cancer, cell-autonomous apoptosis constitutes a common tumor suppressor mechanism, a property which is exploited in cancer therapy [98]. According to this, regrettably, our tested compounds did not induce cell death by apoptosis mechanisms.

DNA damage is detected conducting the Alkaline SCGE assay [99], which is widely used to determine whether cells are undergoing apoptotic and/or necrotic pathways [100]. Apoptosis occurs when treatments induce a TM > 30 (hedgehog pattern), whereas control cells remain lower than 2 (no tails). On the contrary, necrosis shows a short comet-tail pattern [101]. Our results showed that PEP did not exhibit clastogenic activity since the TM values of all assayed concentrations remained in TM values lower than one. This finding means that PEP can be regarded as untreated cells (class 0) from the five TM classes proposed by Fabiani, et al. [102]. FRU (0.57 mg/mL) significantly exerted a clastogenic effect on the HL-60 cell line as this concentration provided a TM value higher than 5 ua, causing damage class II (5–10ua TM) on the HL-60 cell line. DCC caused damage class I (1–5ua TM).

PEP has been demonstrated to be clastogenic, inducing chromosomal aberrations in bone marrow cells of Wistar rats [89] and in the roots of *Allium cepa* [86]. Furthermore, PEP caused a chromosomal break and bridges in *Allium cepa* [86]. This damage caused by PEP could be attributed to their various components, such as caffeine [13,103], and acidity (pH 3.5) since low pH can reduce the metabolic rate and the body’s antioxidant defenses [104]. In other studies, PEP consumption in mice reduced the uterine expression of the FSHR protein; thus, PEP is involved in DNA expression, causing damage to some extent [105]. In addition, 1% FRU did not induce chromosomal aberrations in the wild type brains of *D. melanogaster* [106]. However, Levi and Werman [107] demonstrated that FRU imposed DNA damage and apoptosis in lymphoma cells from mice, which is congruent with our results to some extent. Recently, FRU has been reported to increase the TM in human lymphocytes, causing genotoxicity especially at higher concentrations [108,109]. The lack of in vitro genetic damage could be due to the fact that the assessed concentrations in the comet assay were the three-lowest ones, which are less cytotoxic given that cell viability is roughly 80% in both substances. Furthermore, the results obtained in the comet assay are congruent with those obtained in our DNA fragmentation test. Anyway, the effect of FRU differs in a concentration-dependent manner [71].

Despite the cytotoxic activity shown by both compounds, none of them induced internucleosomal DNA fragmentation and DNA damage at the assayed concentrations; thus, the cell death is not due to apoptotic mechanisms in our HL-60 model, except for the 18 mg/mL FRU. Classic coke and caffeine-free coke were demonstrated to provoke internucleosomal DNA fragmentation at the second-highest concentration in HL-60 cells [13], a similar concentration to FRU assayed in this study. Therefore, FRU could explain the effect of both beverages.

Epigenetics in cancer cells often involves a global reduction in DNA methylation, which activates transposable elements and contributes to genomic instability [110]. Conversely, hypermethylation is closely linked to the inactivation of tumor suppressor genes [111]. In normal somatic cells, repetitive sequences are typically heavily methylated, ensuring global genomic hypermethylation, which suppresses the activity of these transposable elements [40].

Three repetitive sequences frequently studied for their methylation patterns include LINE-1, Alu M1, and Sat-α. LINE-1, a type of long interspersed nuclear element, comprises approximately 17% of the human genome and accumulates in chromosome regions rich in adenine and thymine (AT-rich regions) and areas of low recombination, especially on the X chromosome [112,113]. Alu elements, part of the short interspersed nuclear elements (SINEs) family, constitute about 10% of the human genome and are predominantly found in guanine–cytosine (GC-rich) regions [113,114]. Lastly, Sat-α sequences, also known as satellite alpha repeats, consisting of tandem DNA sequences of 170 base pairs, represent around 5% of human DNA, and are the principal component of centromeric regions [113,115].

Assessing the methylation levels of these repetitive elements—LINE-1, Alu, and Sat-α—has become a useful method for gauging global DNA methylation, given that they collectively account for roughly 32% of the human genome [116].

To our knowledge, this is the first attempt at evaluating the ability of PEP and DCC for modulating the epigenome; thus, there is not any information related to this assay using PEP and DCC on scientific databases. Our results yielded that PEP and DCC globally hypermethylated the three repetitive elements studied in this assay. This hypermethylation could be considered as a benefit since LINE-1 is associated with the C-met oncogene, which would be silenced [117]. In addition, it has been demonstrated that the expression of satellite sequences is associated with hypomethylation triggering cancer cells. Therefore, the methylation process in satellite sequences is a potential mechanism for silencing its satellite expression in transformed cells [118]. This assumption supports the hypothesis that PEP and DCC could be considered as chemopreventive agents that silence the expression of transposons and oncogenes. FRU does not have any influence in these tested repetitive elements. Despite this fact, its benefit on human therapies is not clear and many more research studies should be performed [119]. It is assumed that FRU regulates a number of genes involved in novo lipogenesis and fatty acid oxidation through an epigenetic mechanism in rats, and this effect of FRU may be transmitted from FRU-fed mothers to offspring, despite the removal of FRU from the diet [120].

## 5. Conclusions

In conclusion, the safety of FRU, PEP, and DCC was confirmed as none of these compounds reached their lethal dose 50 (LD_50_) during toxicity tests, nor did they result in mutation rates exceeding the concurrent control in *Drosophila melanogaster*. Additionally, antigenotoxicity assays demonstrated that PEP and DCC protected DNA against oxidative stress caused by hydrogen peroxide in *D. melanogaster*, while PEP alone exhibited protective effects in the antitoxicity assay. FRU notably enhanced both the life expectancy and quality of life of this model organism, whereas PEP was associated with a reduction in its lifespan.

All three substances exhibited chemopreventive properties, with FRU and DCC showing some degree of clastogenic activity in human leukemia cells. Both PEP and DCC influenced the methylation patterns of the HL-60 cell line. Further research is needed to clarify the mechanisms underlying their potential use in gene therapies.

## Figures and Tables

**Table 2:** Toxicity and antitoxicity levels of FRU, PEP, and DCC in *D. melanogaster*.

FRU	Survival		PEP	Survival		DCC (mg/mL)	Survival (%)	
(mg/mL)	(%)		(mg/mL)	(%)				
	Simple	Combined		Simple	Combined		Simple	Combined
	Treatment	Treatment		Treatment	Treatment		Treatment	Treatment
0	100	100	0	100	100	0	100	100
H_2_O_2_	-	49	H_2_O_2_	-	42	H_2_O_2_	-	52.3
0.57	87.7	56	0.8	89.33	50	0.023	91	55
2.25	96	54	3.4	89.33	47	0.09	91	56
4.5	88.35	56	6.87	86 *	47	0.18	89	47
18	100	71 *	27.5	80 *	44	0.75	96	51.65
72	99.34	48.35	110	65.33 *	34	3	94	45

Data are expressed as percentage of survival adults with respect to 300 untreated 72-h-old larvae from three independent experiments. Combined treatments used standard medium and 0.15 M hydrogen peroxide. Asterisks (*) indicate significant differences (one tail) with respect to the untreated control group and the hydrogen peroxide control group (Chi-square value higher than 5.02 [13]). Hyphen (-) means no data.

**Table 3:** Genotoxicity and antigenotoxicity assays of FRU, PEP, and DCC in *D. melanogaster*.

Clones per Wings (Number of Spots) ^1^							
Compound	Wing Number	Small Single Spots (1–2Cells) m = 2	Large Simple Spots (>2 Cells) m = 5	Twin Spots m = 5	Total Spots m = 2	Mann–Whitney Test ^2^	IP (%) ^3^
H_2_O	41	0.147 (6)	0.048 (2)	0	0.195 (8)		
H_2_O_2_ (0.15 M)	40	0.375 (15)	0.05 (2)	0	0.425 (17) +		
Simple Treatment							
FRU (mg/mL)							
2.25	38	0.28 (11)	0.1 (1)	0	0.32 (12) i	λ	
72	33	0.24 (8)	0.03 (1)	0.03 (1)	0.3 (10) i	λ	
PEP (mg/mL)							
3.4	42	0.071 (3)	0	0	0.071 (3) −		
110	44	0.18 (8)	0.022 (1)	0	0.204 (9) −		
DCC (mg/mL)							
0.09	40	0.175 (7)	0.075 (3)	0	0.25 (10) i	λ	
3	36	0.25 (9)	0	0	0.25 (9) i	λ	
Combined Treatment							
FRU (mg/mL)							
2.25	50	0.18 (9)	0.08 (4)	0	0.26 (13) β	ω	
72	40	0.275 (11)	0.125 (5)	0	0.4 (16) β	ω	
PEP (mg/mL)							
3.4	44	0.11 (5)	0.023 (1)	0	0.136 (6) *		68
110	40	0.1 (4)	0	0	0.1 (4) *		76.5
DCC (mg/mL)							
0.09	40	0.05 (2)	0.05 (2)	0	0.1 (4) *		76.4
3	44	0.227 (10)	0.068 (3)	0	0.3 (13) β	ω	

^1^ Statistical diagnosis according to Frei and Wurgler (1988) [41]: + (positive), − (negative), and i (inconclusive) vs. negative control; * (positive) and β (inconclusive) vs. respective positive control; m: multiplication factor. Kastenbaum–Bowman Test without Bonferroni correction, probability levels: α = *β* = 0.05. No. of spots in parentheses. ^2^ Mann–Whitney test was used when appropriate to resolve inconclusive results. Lambda (λ) and Omega (ω) symbols mean that there are not significant differences with respect to the negative and positive control, respectively. ^3^ Inhibition percentage values were included when appropriate.

**Table 4:** Effects of FRU, PEP, and DCC treatments on the *D. melanogaster* mean lifespan and healthspan.

	Mean Lifespan (Days)	Mean Lifespan Difference (%) ^a^	Healthspan (80th Percentile) (Days)	Healthspan Difference (%) ^a^
FRU (mg/mL)				
Control	59.67 ± 2.9	0	32.63 ± 1.48	0
0.57	68.37 ± 2.8 *	14.58	33.66 ± 1.69	3.15
2.25	67.76 ± 2.27	13.5	44.58 ± 3.9	36.62
4.5	73.8 ± 2.35 ***	23.7	41.68 ± 2.81 *	27.73
18	70.6 ± 1.7 **	18.31	46.87 ± 1.87 **	43.64
72	59.334 ± 2.25	−0.6	27.7 ± 3.42	−15.1
PEP (mg/mL)				
Control	64 ± 3.12	0	31.21 ± 2.37	0
0.8	60.5 ± 2.9	−5.5	31.18 ± 1.67	−0.1
3.4	58.2 ± 2.7	−9.1	30.6 ± 1.8	−1.96
6.87	52.3 ± 2.3 ***	−18.3	31.5 ± 1.52	0.92
27.5	52.34 ± 2.8 **	−18.3	27.9 ± 1.05	−10.6
110	51.9 ± 2.7 **	−18.9	28.62 ± 1.13	−8.3
DCC (mg/mL)				
Control	59.67 ± 2.92	0	32.62 ± 1.48	0
0.023	66.4 ± 3.3	11.27	31.62 ± 1.88	−1
0.09	62 ± 2.8	3.9	31.14 ± 1.7	−4.5
0.18	57.2 ± 2.4	−4.2	32.85 ± 1.66	0.7
0.75	68.27 ± 2.26 *	14.41	40.68 ± 2.43	24.7
3	65.14 ± 2.55	9.16	40.23 ± 2.3	23.33

^a^ The difference was calculated by comparing treated flies with the concurrent water control. Positive numbers indicate a lifespan increase and negative numbers indicate a lifespan decrease. Data are expressed as mean value ± SE. * *p* ≤ 0.05, ** *p* ≤ 0.01, *** *p* ≤ 0.001 significances obtained with the log-rank (Mantel–Cox) test.

## Data Availability

The original contributions presented in this study are included in the article. Further inquiries can be directed to the corresponding author.
